# I Just Can’t Get Enough (of Experts): The Numbers of COVID-19 and the Need for a European Approach to Testing

**DOI:** 10.1017/err.2020.41

**Published:** 2020-04-23

**Authors:** Marta MORVILLO

**Affiliations:** * Amsterdam Centre for European Law and Governance, University of Amsterdam; email: m.morvillo@uva.nl.

## Abstract

This article offers a reflection on the testing strategies deployed in the generation of epidemiological data in the European Union (EU). I will argue that, while in the early days of the pandemic, Member States proceeded to testing in a rather scattered way, the shortage of resources seems to have acted as a driver of coordination, which is now increasingly being discussed at EU level. I will examine the legal and institutional framework supporting such embryonic coordination efforts and offer a preliminary assessment of their implications for a European approach to epidemiological knowledge-making.

## I. Introduction: measuring the pandemic between risk assessment and risk management

Among the many things that have been run over, at least temporarily, by the COVID-19 pandemic, one can surely include Michael Gove’s notorious pre-Brexit outburst of “we’ve had enough of experts”. As we keep refreshing news portals’ homepages and look for authoritative voices to help us make sense of the events we are witnessing from our homes, we definitely do not seem to have had enough of experts.

And neither have Heads of States and Governments. The “herd immunity” approach initially proclaimed by the British Prime Minister has been quickly reconsidered after the release of an Imperial College study quantifying the human loss at 260,000 deaths in the absence of containment measures.^1^ References to expert advice as a basis for the actions adopted to face the pandemic have been unanimous and extensive in political leaders’ statements, including those of populist, allegedly anti-elite leaders. While heightened by the emergency, the critical dependence of contemporary decision-making on expert input is of course no news.^2^ After the emergence and consolidation of political movements questioning the very essence of expertise over the last few years,^3^ however, the pandemic is reinstating – and luckily so – the crucial role experts play in informing decisions with deep societal consequences.

What the COVID-19 outbreak also shows us is that expertise in general, and regulatory expertise in particular, is neither monolithic nor acontextual. As most governments invoked the respective expert agencies’ epistemic authority as the basis for risk management measures, such measures have been all but uniform, even across Europe.^4^ With limited knowledge and unprecedented circumstances on a global scale, uncertainties, disagreements and changes in approach are fully understandable, if not unavoidable. We are indeed facing one of Ulrich Beck’s “new modernity” scenarios characterised by radical uncertainty.^5^ What is more relevant in this context, however, is that appeals to expertise have been used to justify measures as different as the total lockdown in Italy and the Swedish no-lockdown approach. Is this only a risk management issue, whereby different political communities (but not yet a European one) reached different conclusions as to the respective weight to be attached to the various societal goods at stake? Or is this also a risk assessment problem, whereby risks have been assessed according to different approaches? The fragmented lines of national borders have quickly thickened not only with regards to free movement of goods and people, but also with regards to the science behind risk management, as in the case of testing, begging the question of what role is there to play for the European Union (EU), not only in coordinating the responses to the emergency, but also in developing a common approach to the generation of the knowledge underpinning decision-making.

Starting from these considerations, in the following paragraphs I offer a reflection on a relatively circumscribed aspect of risk assessment in the COVID-19 pandemic and on a specific type of expert input into the decision-making process: the generation of epidemiological data. As we have sadly become accustomed to daily reports by government agencies and international organisations on the numbers of infected and victims, discussions have sparked over what such numbers represent and how they represent it: the high variation of morbidity and mortality rates across countries and the impact of the number of tests carried out in order to establish them have been some of the most discussed issues so far.^6^ I will argue that, while in the early days of the pandemic, Member States proceeded to testing in a rather scattered way, the shortage of resources seems to have acted as a driver of coordination, which is now increasingly being discussed at EU level. I will examine the legal and institutional framework supporting such embryonic coordination efforts and offer a preliminary assessment of their implications for the development of a European approach to epidemiological knowledge-making.

## II. Comparing epidemiological data in the EU: tests, resources and representation

The global reach of the pandemic has made the comparability of epidemiological data essential. Being able to trace the spread of the disease, its pace and its mortality rate across different countries has proved to be vital to trying to predict its evolution, putting in place adequate containment measures and evaluating their effects. The generation of epidemiological data is located at the crossroads of technical and policy evaluations taking place at a variety of levels. Data collected by healthcare professionals are validated by national laboratories, which report at least daily to the competent national authority, and are then channelled towards the international (World Health Organization (WHO)) and European level (European Centre for Disease Prevention and Control (ECDC)).^7^ Consistency in the data collection is ensured, on the one hand, by the adoption of a uniform “case definition” at WHO level,^8^ which identifies suspect, probable and confirmed cases, and, on the other hand, by the data collection requirements, also established by the WHO.

When considering the comparability of epidemiological data across countries, another relevant aspect is that of the number of tests carried out. The WHO has been clear in stressing that countries should strive to carry out as many tests as possible, especially at the early stages of the epidemic.^9^ Active case finding and contact tracing are considered essential from two points of view: they allow for the early detection and isolation of cases and they widen the information basis on the characteristics of the disease, so as to enable further research.^10^ Notwithstanding the WHO’s unconditional call for extensive testing, not all countries have followed the same approach to the detection of COVID-19 cases. Even within the EU, testing efforts have significantly differed, especially at the beginning of the COVID-19 outbreak. Italy has engaged in widespread testing, until the excessive pressure on testing facilities forced a prioritisation of the most exposed and vulnerable subjects.^11^ The Netherlands has, from the very beginning, announced a “judicious” approach to testing due to the need to ensure the long-term availability of testing material.^12^ Germany is the European country with the largest testing capacity.^13^ The initial approaches to testing may have been the result of different scientific and non-scientific considerations (including different cultural sensitivities towards social distancing, intensity of inter-generational interactions, perception of the disease, etc.). As the spread of the virus advanced throughout Europe, it became clear, however, that Member States have very different testing capacities and mostly struggle to meet the demands of testing kits and personnel. It has also become evident that testing will play a key role in the progressive lifting of the containment measures.^14^

While the role of testing is largely a matter of resources, it also has deep repercussions on the representation of the problems it aims at measuring: the differences in the number of tests carried out from country to country lead to different results in terms of mortality and morbidity, thus opening the way to different risk management scenarios. This potential distortion seems particularly problematic in contexts such as that of the EU, where the deep economic and social integration make it essential to set out a coordinated response in the region. Against this background, the following section will consider the legal and institutional context in which the first steps towards a common approach to testing at European level could be taking place.

## III. Where coordination (could) happen: the European Centre for Disease Prevention and Control, the advisory panel on COVID-19 and the Health Security Committee

### 1. Legal framework and current initiatives

At EU level, there is currently no legal provision setting out common rules on the testing strategies to be pursued in the case of an epidemic. Decision 1082/2013/EU, on serious cross-border threats to health,^15^ and Regulation 851/2004,^16^ establishing the ECDC, both envisage information exchange and reporting duties on Member States and set out a framework for cooperation in epidemiological data collection and exchange. A reference to consultations, with a view to coordinating national capacities as to the monitoring and response to cross-border health threats, is to be found in Article 4 of the 2013 Decision. There is, however, no basis for a centralised or integrated management of the testing capacity of Member States’ laboratories or for a common testing strategy. This comes as no surprise if one considers how closely testing capacity is linked to the allocation of healthcare resources and hence to the discretionary choices Member States are likely to be willing to retain. More generally, it resonates well with the dynamics underpinning the evolution of EU health law. While the EU’s involvement in the area has gradually consolidated and expanded,^17^ its competences remain limited, with public health lying at the core of State-administered welfare – and at the centre of State budgets as well as of social and cultural sensitivities.^18^ Such expansion has therefore been taking place along the sideways of coordination rather than through legislative action, exploiting Article 6(a) of the Treaty on the Functioning of the European Union (TFEU), rather than Article 4(2)(k) TFEU.^19^ The development of a common testing strategy for COVID-19 could be emerging along similar lines.

As already mentioned, Member States have gradually converged towards the need to carry out as many tests as possible, while being faced with a shortage of testing resources. In this context, the Commission has started exploring two complementary courses of action, seeking, on the one hand, to address the lack of testing kits and, on the other hand, to coordinate their deployment. Both strategies are embedded in the 2013 Decision on serious cross-border threats to health. The former has been carried out by the Commission under Article 5 thereof, which envisages the possibility of launching joint procurement procedures in order to purchase medical equipment. The latter has resulted in recommendations for testing strategies addressing how to prioritise testing in case of limited resources^20^ and in a Commission communication containing guidelines on COVID-19 *in vitro* diagnostic tests.^21^ The recommendations start by acknowledging that the situation varies across Member States (eg with regards to the presence of both community transmission and local epidemics) and sets out common criteria on how to prioritise the different categories of patients. The guidelines, on the other hand, aim at ensuring that safe and effective devices for COVID-19 testing are available in the EU and provide guidance as to what information tests can deliver and as to their performances. Among other things, they recall the actions undertaken by the Commission, including information sharing on national actions, issuing of guidance on medical devices’ conformity assessment and performance criteria and the distribution of quality assessment materials among European laboratories.

### 2. Institutional framework

While having a basis in Article 4(1)(d) and Article 11(1)(a) of the 2013 decision, the development of such recommendations and guidelines seems to be embedded in a broader process of coordination that sees an interplay of political and technical actors operating at the crossroads of national and European institutions. In particular, discussions on possible common approaches to testing, including those set out in the recommendations and guidelines, have been taking place in the context of the ECDC, of the COVID-19 expert panel set up by the Commission and of the Health Security Committee (HSC), and are still ongoing at the time of writing. While the first two are typically technical bodies, one permanent (the ECDC) and one ad hoc (the expert panel), the third is political in nature.

The ECDC is an agency primarily entrusted with risk assessment tasks, and in particular with the identification, assessment and communication of “current and emerging threats to human health from communicable diseases”.^22^ It coordinates the Early Warning Response System,^23^ as well as data collection, validation, analysis and dissemination, with the goal of promoting synergy and avoiding duplication.^24^ The ECDC has no decision-making powers,^25^ but provides scientific opinions and technical assistance to the Commission and Member States. It plays a crucial role in coordinating Member States’ actions in the collection of epidemiological data, in particular by ensuring homogeneity in the type of information collected. In the context of the COVID-19 crisis, the ECDC has, *inter alia*, drafted the proposal for common testing strategies, which has then been discussed by the Commission’s expert panel and by the HSC.

The second actor involved in the efforts to coordinate testing strategies at EU level is the COVID-19 expert panel assisting the Commission in elaborating a response to the health crisis. It is an ad hoc advisory body set up as an expert group by the Commission in March 2020, with the task of formulating “science-based EU response guidelines and coordinate risk management measures”.^26^ The panel is chaired by the Commission President and by the Commissioner for health and food safety and is composed of scientists appointed in a personal capacity among persons with acknowledged expertise areas such as epidemiology, virology, public health and crisis management. The ECDC, the European Medicines Agency (EMA) and the Emergency Response Coordination Centre (ERCC) act as observers. Testing strategies have been one of the recurrent topics of the panel meetings so far, especially since the scarcity of testing resources has become evident.^27^ Based on the ECDC proposals for common testing strategies, the expert panel has advised to “raise the issue of testing strategies with Member States and to quickly work on EU guidelines”;^28^ it also stressed that “testing would be one of the key points of any future exit strategy and a coordinated approach to testing capacity would be required”, depending also on the availability of rapid testing methods.^29^

The third avenue where different testing strategies and possible common approaches are discussed is the HSC. The HSC is established under Article 17 of Decision 1082/2013 and is composed of health ministry representatives of Member States. It is chaired by a Commission representative (normally from Directorate-General for Health and Food Safety, unit C3 on crisis management and preparedness in health) with the participation, as observers, of representatives of the European Economic Area members and of other bodies such as the EMA, the ECDC and the WHO. The HSC therefore has a political connotation, as opposed to the primarily technical nature of the ECDC and the Commission expert panel. In particular, its mission is to reinforce the coordination among Member States both as to preparedness and as to the response to serious cross-border threats. It has been noted that HSC enjoys a high degree of “authoritative strength, or, [in other words] it was able to present Member States with a ‘moral bindingness’”.^30^ In the context of the COVID-19 crisis, it has repeatedly discussed issues related to testing,^31^ and in particular how to address the shortage of testing material and “aspects influencing changes to strategies in view of the EU recommendations”.^32^

### 3. Future initiatives and the politics of testing

If a common approach to testing will emerge in the coming weeks or months, it will most probably be through the HSC: it is here that the Commission, with the scientific advice of the ECDC and the ad hoc expert panel, can best exert political pressure on Member States towards a coordinated exit strategy, and it is here that politically accountable actors (national health ministry representatives) can confer such a strategy a relatively solid legitimacy vis-à-vis national constituencies.^33^ In its communication, the Commission has already outlined possible courses of action in order to foster testing strategy coordination “as far as this is appropriate”. These range from facilitating the discussion of national testing strategies (and in particular of how to account for the different purposes of the various types of tests and for the different contexts where they are deployed), to assistance through a centralised overview of available information on test performance. Importantly, the Communication also mentions the establishment of a network of reference laboratories across the EU in order to facilitate not only exchanges of information and skills, but also the development of common methodologies and the distribution of resources (particularly control samples, tests and reagents) according to EU-wide supply and demand. The network will be coordinated by the Commission itself, in cooperation with the Member States and in consultation with the ECDC.

Throughout the communication, it is possible to trace a tension between the Commission’s urge for a coordinated approach and the awareness of Member States’ possible resistance. More EU-level coordination might indeed be perceived by Member States as an intrusion into a highly sensitive field such as public health, all the more in the context of a health crisis that is exacerbating social and political conflicts at both national and EU level. Against this background, the Commission’s appeal for solidarity appears two-fold. On the one hand, it sounds like an exhortation to learn from the early days of the COVID-19 crisis (Member States “should show solidarity in making arrangements for the fair distribution of available stocks and laboratory equipment to where they are most needed”^34^). On the other hand, it reveals the Commission’s concern for the transition towards a post-COVID-19 Europe where “coordination of national strategies will be indispensable”,^35^ and where, conversely, the prevalence of national interests over coordination could not only be detrimental in terms of public health and economic recovery, but also politically fatal.

## IV. Conclusion: from pooling resources to a European approach to testing?

In his 1999 book *Powers of Freedom*, Nikolas Rose speaks of “political numbers” referring to the “reciprocal and mutually constitutive” relation between numbers and politics.^36^ Such a relation manifests itself in (at least) two directions. On the one hand, the political nature of numbers emerges in their very genesis. Discretion is “implicit in the choice of what to measure, how to measure it, how often to measure it, and how to present and interpret the results”.^37^ On the other hand, it is manifest in the circumstance that, by portraying and simplifying reality, numbers allow for different courses of action. Numbers “actually constitute the domains they appear to represent; they render them representable in a docile form – a form amenable to the application of calculation and deliberation”.^38^ They therefore come to be closely linked not only to the representation (and problematisation) of facts, but also to the determination of the available courses of action.

In this sense, a political dimension is also present in the generation of epidemiological data, and particularly in the definition of testing strategies in the context of the COVID-19 crisis. Attempts at coordination are taking place at EU level through a dialogue between technical (ECDC and expert panel) and political (HSC) actors against a background of limited EU competences and conflicting national interests.

Testing is, in fact, not merely a technical issue, but is rather located at the crossroads of scientific and political considerations, involving, for example, “testing availability capacities, as well as identification of risk groups or risk areas across Member States”:^39^ choices also need to be made in how to generate epidemiological data. Accordingly, such data, and in particular the number of tests carried out, have a close relationship with discretion: discretionary considerations, ultimately based on resources’ availability and, at least to an extent, on value choices, have a significant bearing on their genesis; discretionary are also – and more obviously – the risk management measures adopted on the basis of the conclusions drawn from them.

The question is then if and to what extent a European dimension will emerge in the representation and problematisation of the pandemic. While at the beginning of the crisis Member States seem to have proceeded in a rather disorderly way, as soon as the resource constraints became apparent, efforts have been put in place towards a common approach to testing, including both the procurement and deployment of testing resources. Such efforts will probably continue, as testing will be increasingly crucial in the recovery phase. It is, however, highly unlikely that a fully-fledged common approach to testing will be developed, also in consideration of the limited EU competences and of the dynamics characterising the evolution of EU health law.^40^ What seems more likely is instead a consolidation of the already ongoing coordination efforts, in particular with regards to access to testing resources through public procurement and, possibly, through internal market-based initiatives (eg to facilitate the circulation of people and goods in the aftermath of the acute phase of the crisis). What seems crucial, however, is that the problematisation of the health crisis (including its quantitative aspects) is not left exclusively to haphazard Member States’ approaches, increasing the risk of a fragmented representation of reality and, likely, of a fragmented response.

## Footnotes

1 S Boseley, “New Data, New Policy: Why UK’s Coronavirus Strategy Changed”, *The Guardian*, 16 March 2020 <https://www.theguardian.com/world/2020/mar/16/new-data-new-policy-why-uks-coronavirus-strategy-has-changed> (last accessed 16 April 2020). The Imperial College report is available at <https://www.imperial.ac.uk/media/imperial-college/medicine/sph/ide/gida-fellowships/Imperial-College-COVID19-NPI-modelling-16-03-2020.pdf> (last accessed 16 April 2020).

2 S Jasanoff, *The Fifth Branch: Science Advisers as Policymakers* (Cambridge, MA, Harvard University Press 1990).

3 T Nichols, *The Death of Expertise: The Campaign against Established Knowledge and Why It Matters* (Oxford, Oxford University Press 2017).

4 Especially in the early days of the pandemic, before most European States – with a few exceptions such as Sweden – converged towards a lockdown. For an overview of the different measures adopted across Europe, see “How Do Coronavirus Containment Measures Vary across Europe?” *The Guardian*, 16 March 2020 <https://www.theguardian.com/world/2020/mar/12/how-do-coronavirus-containment-measures-vary-across-europe?CMP=Share_iOSApp_Other> (last accessed 16 April 2020).

5 U Beck, *Risk Society: Towards a New Modernity* (Thousand Oaks, CA, Sage 1992). See also A de Ruijter and M Weimer, “COVID-19 Crisis: Lessons from Risk Regulation for EU Leaders”, *EU Law Live*, 13 March 2020 <https://eulawlive.com/weekend-edition> (last accessed 16 April 2020).

6 See, eg, K Bennhold, “A German Exception? Why the Country’s Coronavirus Death Rate Is Low”, *The New York Times*, 4 April 2020 <https://www.nytimes.com/2020/04/04/world/europe/germany-coronavirus-death-rate.html> (last accessed 16 April 2020).

7 At the time of writing, there is a variety of sources collecting and elaborating epidemiological data into comparable formats. Globally, the three most authoritative are the Johns Hopkins University database: <https://gisanddata.maps.arcgis.com/apps/opsdashboard/index.html#/bda7594740fd40299423467b48e9ecf6> (last accessed 16 April 2020); the World Health Organization: <https://www.who.int/emergencies/diseases/novel-coronavirus-2019/situation-reports> (last accessed 16 April 2020); and the European Centre for Disease Prevention and Control: <https://qap.ecdc.europa.eu/public/extensions/COVID-19/COVID-19.html> (last accessed 16 April 2020). For an overview of the differences among the three databases, see H Ritchie, E Ortiz-Ospina, M Rose and J Hasell, “COVID-19 Deaths and Cases: How Do Sources Compare?” *Our World in Data*, 19 March 2019 <https://ourworldindata.org/covid-sources-comparison> (last accessed 16 April 2020).

8 WHO, “Global Surveillance for COVID-19 Caused by Human Infection with COVID-19 Virus”, interim guidance 20 March 2020, 1–2. The WHO definition has been adopted by the ECDC: <https://www.ecdc.europa.eu/en/case-definition-and-european-surveillance-human-infection-novel-coronavirus-2019-ncov> (last accessed 16 April 2020).

9 WHO, “Critical Preparedness, Readiness and Response Actions for COVID-19”, interim guidance 22 March 2020.

10 WHO, supra, note 8. The WHO is, however, aware of the limited resources and of the subsequent possible need to prioritise some subjects over others when it comes to testing, and it sets out criteria for prioritisation.

11 Ministero della Salute, “Pandemia di COVID-19 – Aggiornamento delle indicazioni sui test diagnostici e sui criteri da adottare nella determinazione delle priorità. Aggiornamento delle indicazioni relative alla diagnosi di laboratorio”, 4 April 2020.

12 See National Institute for Public Health and the Environment, “Policy on Testing for Novel Coronavirus Disease (COVID-19)” <https://www.rivm.nl/en/novel-coronavirus-covid-19/testing-policy> (last accessed 16 April 2020). See also E van Ark and J-H Strop, “Roche Releases Recipe after European Commission Considers Intervention Due to Lack of Coronavirus Tests”, *Follow the Money*, 27 March 2020 <https://www.ftm.nl/artikelen/roche-releases-recipe-after-public-pressure-while-european-commission-considers-intervention-due-to-coronavirus-test> (last accessed 16 April 2020).

13 For an overview of the varying testing capacities across Europe, see ES Nicolas, “EU Countries Unable to Follow WHO’s Call for Mass Testing”, *EUObserver*, 20 March 2020 <https://euobserver.com/coronavirus/147830> (last accessed 16 April 2020).

14 European Commission, “Communication from the Commission, Guidelines on COVID-19 *In Vitro* Diagnostic Tests and Their Performance”, C(2020) 2391 final, 15 April 2020. See also R Baldwin, “COVID-19 Testing for Testing Times: Fostering Economic Recovery and Preparing for the Second Wave’ *VOX CEPR Policy Portal*, 26 March 2020 <https://voxeu.org/article/testing-testing-times> (last accessed 16 April 2020).

15 Decision No 1082/2013/EU of the European Parliament and of the Council of 22 October 2013 on serious cross-border threats to health and repealing Decision No 2119/98/EC [2013] OJ L293/1 (Decision on serious cross border threats to health). See F Brito Bastos and A de Ruijter, “Break or Bend in Case of Emergency?: Rule of Law and State of Emergency in European Public Health Administration” (2019) 10 European Journal of Risk Regulation 610, 624 et seqq.

16 Regulation (EC) No 851/2004 of the European Parliament and of the Council of 21 April 2004, establishing a European centre for disease prevention and control [2004] OJ L142/1 (ECDC regulation). For the process leading to the establishment of the ECDC, see MR Roberts, “The European Centre for Disease Prevention and Control: Science and Political Integration in Europe”, *UCL STS Observatory*, 2 August 2013 <https://blogs.ucl.ac.uk/sts-observatory/2013/08/02/the-european-centre-for-disease-prevention-and-control-science-and-political-integration-in-europe> (last accessed 16 April 2020); SL Greer, “The European Centre for Disease Prevention and Control: Hub or Hollow Core?” (2012) 37 Journal of Health Politics, Policy and Law 1001.

17 See Art 168, in particular (2), and Art 169 TFEU. See also A de Ruijter, *EU Health Law and Policy: The Expansion of EU Power in Public Health and Health Care* (Oxford, Oxford University Press 2019).

18 AM Pacces and M Weimer, “From Diversity to Coordination: A European Approach to COVID-19” (2020) European Journal of Risk Regulation, forthcoming.

19
ibid. On the potential of the EU framework on health emergencies, see also KP Purnhagen, A de Ruijter, ML Flear, TK Hervey and A Herwig, “More Competences than You Knew? The Web of Health Competence for Union Action in Response to the COVID-19 Outbreak” (2020) European Journal of Risk Regulation, forthcoming. On its limits, see A Renda and RJ Castro, “Towards Stronger EU Governance of Health Threats after the COVID-19 Pandemic’ (2020) European Journal of Risk Regulation, forthcoming.

20 European Commission, “COVID-19, EU Recommendations for Testing Strategies”, 18 March 2020 <https://ec.europa.eu/info/sites/info/files/covid19_-_eu_recommendations_on_testing_strategies_v2.pdf> (last accessed 16 April 2020).

21 European Commission, supra, note 14. See also European Commission, “Testing Kits for COVID-19: What Is the EU Doing?”, 15 April 2020 <https://ec.europa.eu/health/sites/health/files/preparedness_response/docs/testingkits_factsheet.pdf> (last accessed 16 April 2020).

22 Art 3(1) ECDC regulation.

23 Arts 8–11, Decision on serious cross border threats to health.

24 Art 11(1) ECDC regulation.

25 Art 3 ECDC regulation.

26 The expert panel has been established with Commission Decision of 16 March 2020 setting up the Commission’s advisory panel on COVID-19, C(2020) 1799 final.

27 See, eg, the expert panel meetings of 12 March 2020, 15 March 2020 and 25 March 2020.

28 Expert panel meeting of 15 March 2020.

29 Expert panel meeting of 25 March 2020.

30 A de Ruijter, supra, note 17, 137.

31 See the HSC meetings of 31 January 2020, 4 February 2020, 25 March 2020, 30 March 2020 and 15 April 2020.

32 HSC meeting of 25 March 2020.

33 See also AM Pacces and M Weimer, supra, note 18.

34 European Commission, supra, note 14.

35
ibid.

36 N Rose, *Powers of Freedom: Reframing Political Thought* (Cambridge, Cambridge University Press 1999) pp 197–98.

37
ibid, 198, quoting W Alonso and P Starr (eds), *The Politics of Numbers* (New York, Russell Sage Foundation 1987) p 3.

38 N Rose, supra, note 36, 198.

39 HSC meeting of 25 March 2020.

40 However, proposals suggesting the development of EU-level testing have also been made: D Gros, “Creating an EU ‘Corona Panel’: Standardised European Sample Tests to Uncover the True Spread of the Coronavirus”, *VOX CEPR Policy Portal*, 28 March 2020 <https://voxeu.org/article/standardised-european-sample-tests-uncover-true-spread-coronavirus> (last accessed 16 April 2020).

